# Adherence to Caloric and Protein Recommendations in Older Hemodialysis Patients: A Multicenter Study

**DOI:** 10.3390/nu14194160

**Published:** 2022-10-07

**Authors:** Sylwia Czaja-Stolc, Ewelina Puchalska-Reglińska, Sylwia Małgorzewicz, Marta Potrykus, Małgorzata Kaczkan, Aneta Kałużna, Zbigniew Heleniak, Alicja Dębska-Ślizień

**Affiliations:** 1 Department of Clinical Nutrition, Medical University of Gdansk, 80-211 Gdańsk, Poland; 2 Dialysis Unit, 7th Navy Hospital in Gdansk, 80-305 Gdańsk, Poland; 3 Department of General, Endocrine and Transplant Surgery, Medical University of Gdansk, 80-211 Gdańsk, Poland; 4 Department of Nephrology, Transplantology and Internal Medicine, Medical University of Gdansk, 80-952 Gdańsk, Poland

**Keywords:** chronic kidney disease, hemodialysis, dietary assessment, nutritional status, malnutrition

## Abstract

Hemodialysis (HD) patients are characterized by malnutrition, which adversely affects their survival. The development of malnutrition is influenced, among other factors, by improper diet and the advanced age of patients. The study aimed to assess the nutritional status and adherence to dietary recommendations among older patients. The multicenter study included 179 stable HD patients. The nutritional status was assessed by a 7-point Subjective Global Assessment (SGA). Anthropometry and body composition was analyzed. The diet was assessed based on the 3-day food diary and the Food Frequency Questionnaire with 6 answers (FFQ-6). Blood laboratory tests were performed. Based on the 7-point SGA, malnutrition was diagnosed in 38.5% of HD patients. The decreased content of the muscle tissue (LTI < 14 kg/m^2^) was observed in 70.4% of the examined patients and the decreased concentration of s-albumin was observed in 44.1% of patients. Older patients had significantly lower LTI. 26% of patients consumed less than 25 kcal/kg body weight and less than 0.8 g protein/kg body weight. Older patients’ diets contained significantly fewer calories. There were significant differences between nutrient intake on a weekday with dialysis, a weekday without dialysis, and a weekend day without dialysis. The lowest intake of nutrients was observed on the day of dialysis. Nutritional education and the determination of whether food is permitted during hemodialysis are necessary to improve patients’ nutrition.

## 1. Introduction

Chronic kidney disease (CKD) is a great contributor to morbidity and mortality from non-communicable diseases. Over the last thirty years, the global all-age mortality rate from CKD has grown to 41.5% [1]. Renal replacement therapy using dialysis is the option to treat end-stage kidney disease (ESKD) and prolong survival [2]. Although dialysis is an effective treatment option for CKD, it may be an independent factor that worsens the nutritional status of patients. Protein-energy wasting (PEW) and poor nutritional status are common and strong predictors of poor outcomes in patients undergoing hemodialysis (HD) treatment [3]. 

Along with the elongation of life and aging of society, the number of elderly people in the population of HD patients increases. In Poland, in 2020, 18,847 patients were hemodialysed, 58% of whom were 65 years old or older. In that year, 4684 patients started renal replacement therapy with hemodialysis or peritoneal dialysis, 65% of whom were elderly [4]. Aging, directly and indirectly, influences the patients’ nutrition, and thus their nutritional status is affected [5]. 

As a person ages, the composition of the body changes—the mass of adipose tissue increases, and muscle tissue decreases. Age-related muscle loss is a gradual process that usually begins after the third decade of life and intensifies after the age of 50 [6].

According to the current nutritional guidelines published in 2020 by Kidney Disease Outcomes Quality Initiative (KDOQI) [7], the protein intake of HD patients should be 1–1.2 g of protein/kg of body weight, and the caloric content of the diet 25–35 kcal/kg of body weight. The International Society of Renal Nutrition and Metabolism (ISRNM) claims that less than 0.8 g of protein/kg of body weight intake and less than 25 kcal/kg of body weight intake are insufficient. Such a diet, even for a short time, can lead to the development of malnutrition [8].

Dialysis treatment causes a loss of proteins and amino acids, which contributes to muscle proteolysis and aggravation of malnutrition. Inflammation occurs in HD patients, which, triggering insulin resistance, also contributes to proteolysis. In turn, insufficient dialysis treatment causes metabolic acidosis and uremia, which affect the altered taste perception and decreased appetite, which reduces food intake [9]. 

It has been observed that the diet of a large percentage of patients does not comply with the recommendations of scientific societies. In an international study, 55% of patients consumed less than 30 kcal/kg body weight and 33% less than 1.1 g protein/kg body weight [10]. According to Burrowes et al., patients consumed fewer calories on dialysis days than on non-dialysis days [11]. In turn, there is research that indicates that hemodialysis patients have lower protein and energy intake on non-dialysis days [12]. These reports stand in opposition to themselves. Available scientific evidence is sometimes ambiguous and inconsistent. Therefore, this work aims to determine whether hemodialysis patients consume less energy and protein on dialysis days than on non-dialysis days and whether the patient’s age affects their nutritional status and diet. A dialysis session itself increases this group of patients’ energy and protein requirements. Insufficient consumption on dialysis days can lead to malnutrition or aggravate existing poor nutritional status. It is essential to check that patients are covering their daily nutrient requirements to introduce early intervention, if necessary. A detailed assessment of the patient’s nutrition will help to identify modifiable factors that may lead to an impaired nutritional status and design the optimal strategy to prevent it. For this purpose, a multicenter study was conducted in which the nutritional status and diet of hemodialysis patients were analyzed. The nutrition in older patients and on days with and without dialysis was compared. Adherence to protein and caloric recommendations in HD patients was assessed.

## 2. Materials and Methods

### 2.1. Study Design

**Figure 1:**
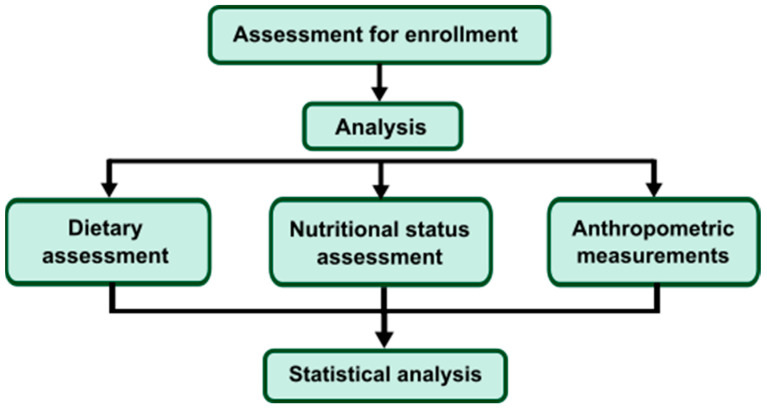
Scheme of the study.

The study was a 3-year, cross-sectional, multicenter study. The study aimed to assess the adherence to the nutritional recommendation by HD patients, compare whether the nutritional status and diet differed in older hemodialysis patients, and check whether the amount of energy and protein in the diet on dialysis days was the same as on days without dialysis. 179 patients in stable clinical conditions were included in the study. Anthropometric measurements and nutritional status assessments using a 7-point Subjective Global Assessment (7-point SGA) were performed on all patients. Each participant was explained how to complete the 6-response Food Frequency Questionnaire (FFQ-6) and the 3-day food diary. The study scheme is shown in Figure 1.

The study obtained the consent of the Independent Bioethical Committee of the Medical University of Gdańsk (NKBBN/417/2015-ST-48, NKBBN/343/2018, NKBBN/613/2020).

### 2.2. Study Population

The study included 179 patients with the 5th stage of CKD treated with maintenance hemodialysis. The patients were undergoing hemodialysis sessions at three dialysis stations located in Poland in the Pomeranian Voivodeship (at University Clinical Centre in Gdańsk, 7th Naval Hospital in Gdańsk, and Diaverum in Kościerzyna). The inclusion criteria were as follows: age over 18 years, minimum 3-month hemodialysis period, and informed and written consent to participate in the study. Patients who had been treated by hemodialysis for less than 3 months, those with cognitive impairment, and with acute diseases were excluded.

All patients included in the study were treated by high-flux hemodialysis, with FX dialyzers with an effective surface area from 1.4–2.2 m^2^. Dialysis sessions were 3 times per week per average 4 h (in the range of 3–5 h). The blood flow was in the range of 300–350 mL/h. The adequacy of dialysis (Kt/V) was 1.6 ± 0.35. All participating centers did not provide meals during the dialysis session.

### 2.3. Nutritional Status

Body mass was tested twice, before and after hemodialysis. A mechanical column scale with a stadiometer was used to measure body weight (kg) and height (meters). Body mass index (BMI) was calculated as the body weight after hemodialysis divided by the square of body height. Nutritional status was assessed with the 7-point SGA which considers the patient’s interview and physical examination. Based on the 7-point SGA score, patients are classified as well-nourished if they receive 6–7 points and malnourished if they receive ≤5 points.

In this study, body composition analysis was performed by electrical bioimpedance using the Fresenius Medical Care Body Composition Monitor 15 min after hemodialysis. Overhydration (OH), total body water (TBW), extracellular water (ECW), intracellular water (ICW), fat tissue index (LTI), lean tissue mass (LTM), lean tissue index (LTI), and body cell mass (BCM) were assessed.

### 2.4. Dietary Assessment

Dietary intake was performed based on a 3-day food diary including a day with dialysis, a day without dialysis, and a weekend day without dialysis. Before starting the food diary, the dietitian instructed patients on how to record the meals they eat. They were asked to write down the weight of consumed meals and drinks. After preparing the diary, the dietitian checked the correctness of its preparation, validated it based on a 24 h diet recall, and asked questions about the preparation of meals. The content of energy and nutrients was estimated based on a specialist diet program (Aliant, Poland), including Polish and the United States Food Composition databases. The ratio of energy and protein consumption to body weight was used for actual body weight in patients with a BMI < 25. For patients with BMI > 25, the ideal body weight is calculated based on the Broca formula.

The FFQ-6 questionnaire validated for the Polish population was used to collect information on the frequency of consumption of 62 assortment groups of products, representing 8 main food groups. Patients had a choice of 6 categories of food consumption frequency: (1) never or almost never, (2) once in a month or less, (3) several times a month, (4) several times a week, (5) daily, (6) several times a day [13].

### 2.5. Biochemistry

The blood samples for tests were collected in the fasting state by qualified nurses before and after the hemodialysis session. The following parameters were determined: blood morphology, serum calcium concentration, phosphorus, parathyroid hormone (PTH), CRP, creatinine, blood urea nitrogen (BUN) (before and after HD), transferrin, ferritin, albumin, and lipid profile. Biochemical parameters were estimated by routine laboratory procedures. Normalized protein catabolic rate (nPCR) was calculated based on Daugirdas’ formula [14].

### 2.6. Data Collection

Sex, age, dialysis vintage, and the cause of kidney failure were obtained by individual medical history and access to medical records. 

### 2.7. Statistical Analysis

The statistical analysis was performed with the use of Microsoft Office Excel 365 and Statistica 13.3 by StatSoft Poland. The variables were assessed for the compliance of their distributions with the normal distribution by means of the evaluation of histograms and the Shapiro-Wilk test. The differences between the two groups were calculated by Student’s *t*-tests or by U Mann–Whitney tests, depending on the distribution. The Friedman ANOVA test was used for multiple paired samples. Chi^2^ Pearson’s test was carried out to evaluate the association between categorized variables. The correlation analysis of variables was performed using the Spearman or Pearson method. The level of statistical significance in the study was *p* < 0.05.

## 3. Results

### 3.1. Characteristics of the HD Patients

The baseline characteristics of 179 hemodialysis patients are presented in Table 1. The most common causes of CKD were glomerulonephritis, polycystic kidney disease, diabetes, or the etiology was unknown. Ultrafiltration, dialysis vintage, Kt/V, and nPCR were similar in both groups. Patients were divided into two groups according to age as presented in Table 1.

### 3.2. Nutritional Status

On the basis of World Health Organization (WHO) classifications [15], 7.3% of the HD patients presented underweight (BMI < 18.5), 41.9% of the subjects had a normal body weight (BMI 18.5–24.99), 32,4% had overweight (BMI 25–29.99), and 18.4% were obese (BMI > 30). Based on the ISRNM criteria regarding the recommended BMI [8,16], 35.2% were malnourished (BMI < 23). In the group of patients under 65 years of age, excessive weight was found in 43.2% of the group, while in the group of older patients it was 56.1%.

Based on the 7-point SGA, PEW was diagnosed in 38.5% of HD patients (SGA ≤ 5). The prevalence of PEW according to 7-point SGA did not differ significantly between the older patients and those under 65 years of age. The decreased lean tissue index (LTI) <14 kg/m^2^ was observed in 70.4% of the examined patients. Among older people, the reduced LTI was found in 75% of respondents, and younger people in 63%. It was observed that among older patients the incidence of reduced LTI was significantly higher than in younger patients (*p* < 0.01). There was a significant correlation between the 7-point SGA and body weight (*p* < 0.05; R = 0.33), BMI (*p* < 0.05; R = 0.30). The nutritional status assessment results are presented in Table 2.

### 3.3. Dietary Assessment

#### 3.3.1. Energy Intake

Finally, for analysis was used 118 correctly completed the 3-day food diary. In the study population, the average caloric intake was lower than recommended by the nephrological society in 66.9% of the respondents [7]. 44.9% of the elderly and 22% of the younger population consumed less than 25 kcal/kg. Multivariate regression model confirm that energy consumption is lower in older adults (B = −0.35, standard error = 0.09, β = −0.18, *p* < 0.01). There were significant differences between caloric intake on a weekday with dialysis, a weekday without dialysis, and a weekend day without dialysis (Table 3). The lowest caloric intake was observed on the day of dialysis. The supply of calories on a weekday with dialysis, a weekday without dialysis, and the average caloric content of the diet were considerably higher among men than among women (*p* < 0.05). There were no significant differences in the caloric content of the diet in well-nourished and malnourished patients. The caloric content of the diet on the dialysis day, and the non-dialysis day were significantly higher among younger people than in the elderly (*p* < 0.01). There was a significant negative correlation between the calorific value of the diet each day and age (*p* < 0.05; R = −0.28).

#### 3.3.2. Protein Intake

The mean dietary protein content/kg body weight in 59.3% of patients was below the recommended level [16]. 28% of patients consumed less than 0.8 g of protein/kg of body weight. 14.4% were older and 13.6% were younger people. The protein consumption of the elderly was lower, but the difference was not statistically significant. The mean nitrogen supply was 10.5 ± 3.52 g and the energy to nitrogen ratio was 139.2 ± 33.3 kcal/g N. There were noteworthy differences between protein intake on a weekday with dialysis, a weekday without dialysis, and a weekend day without dialysis (Table 4). The lowest protein intake was observed on the day of dialysis. Significantly higher protein intake was observed on all days among men compared to women (*p* < 0.05). The mean protein intake was substantially higher among the well-nourished patients (*p* < 0.05). There was a notable correlation between the LTI value and the caloric content of the diet on the weekday with dialysis (*p* < 0.05; R = 0.26), the weekday without dialysis (*p* < 0.05; R = 0.27), the mean caloric content of the diet (*p* < 0.05; R = 0.26), and the protein content in the diet on the weekday with dialysis (*p* < 0.05; R = 0.23).

A correlation between the average caloric content of the diet and average protein intake (*p* < 0.5; r = 0.82) was observed. Despite the high correlation, 39.8% of people consumed an adequate amount of protein but not enough calories, which is also a negative risk factor for the development of malnutrition. 27.1% of patients did not eat enough protein and calories (14.4% of which were elderly).

#### 3.3.3. FFQ-6

Due to the analysis of the FFQ-6 questionnaire, the eating habits of 179 HD patients could be observed. The frequency of consumption of selected food products from each of the eight food groups included in the questionnaire is presented in Table 5. The most often consumed products were refined bread, butter, vegetables, and fruit. The least frequent were venison, energy drinks, vodka, and avocado.

### 3.4. Biochemical Parameters

The mean albumin level was 36.1 ± 6.4 g/L. According to the ISRNM criteria, a decreased concentration of albumin (<38 g/L) was observed in 44.1% of the examined patients. Correlation was observed between albumin level and hemoglobin (*p* < 0.05; R = 0.23). There was a significant correlation between protein intake on the day of dialysis and BUN concentration before (*p* < 0.05; R = 0.26) and after HD (*p* < 0.05; R = 0.25). Correlation between transferrin and 7-point SGA among elderly was observed (*p* < 0.05; R = 0.24). The results of laboratory tests are presented in Table 6.

There was a significant correlation between albumin level and the frequency of consumption of cheese (*p* < 0.05; R = 0.37) and red meat (*p* < 0.05; R = 0.22). There was a significant correlation between protein intake on the day of dialysis and BUN concentration before (*p* < 0.05; R = 0.31) and after HD (*p* < 0.05; R = 0.25). There was a negative correlation between the age of the patients and the mean caloric content of the diet (*p* < 0.5; R = −0.25). The relationship between nutritional status, body composition, and biochemical parameters is presented in Table 7.

## 4. Discussion

Despite the long-standing nutritional recommendations that have been updated in 2020, low protein and caloric intake is still a common problem among HD patients. In our test group, 27.1% of people consumed extremely little protein and calories. the elderly had significantly lower caloric intake. 39.8% of respondents consumed the right amount of protein, but too few calories, which is an unfavorable factor in the development of malnutrition and mortality [17]. In such a situation, the protein is used as an energy substrate. Although the patients who qualified for the study had the opportunity to benefit from a dietary consultation, the consumption was too low.

Protein consumption among the women in our study was 0.49–1.68 g/kg/day and 0.51–1.91 g/kg/day among men. In 2000 Rao et al. showed that 0.6 g/kg/day of protein intake produces a neutral or negative nitrogen balance, and 0.85 g/kg/day may be unsafe. It seems that no more recent publications investigating the nitrogen balance in hemodialysis patients were published. Patients who had less than 0.6 g/kg/day of protein had a negative nitrogen balance and could develop malnutrition [18]. It is recommended to provide 100–150 kcal of non-protein energy for every gram of nitrogen. If the amount of energy is lower, the protein will be used as an energy source [19]. In the examined group, the amount of non-protein energy to nitrogen ratio was 139.2 ± 33.3 kcal/g N, which is in line with the recommendations.

The findings of this study confirm that hemodialysis patients‘ energy and protein intake are lower on dialysis days than on non-dialysis days. Similar results were obtained in other studies [5,11]. We have specifically focused on the elderly group, as the percentage of these patients has increased in dialysis centers in recent decades. It is a group that is particularly vulnerable to malnutrition due to the changes resulting from aging. In the aging process, physiological changes that lead to a reduction in daily energy requirements are observed. The alternations in the digestive tract occur and cause difficulties in meeting nutritional needs. Saliva production and the number of digestive enzymes contained in it are reduced. Atrophic changes appear in the gastrointestinal mucosa. The peristaltic efficiency of the gastrointestinal tract is worsened by the reduction of muscle mass, which in turn can lead to constipation or diarrhea. In the elderly, diseases that have a decisive influence on the appetite often coexist, such as gastroesophageal reflux disease, gastric ulcer, and duodenal ulcer disease. In the stomach, the secretion of gastric juice is reduced. The liver function deteriorates, and the activity of pancreatic enzymes decreases, which led to decreased tolerance of the meal. Physiological changes also affect the mouth and teeth. Missing teeth or pain in the oral cavity impairs the fragmentation of food and may result in the elimination of certain products from the diet. Another cause of the insufficient supply of nutrients is the disturbance of the perception of sensory stimuli. The sense of smell and taste is impaired, so the patient does not experience the pleasure of eating or uses excessive amounts of salt and sugar to increase the palatability of the food [20]. Additionally, taking multiple medications, which is common in older people, can reduce appetite as well as impair the sense of taste. Drug-nutrient interactions can impair the absorption of nutrients. The other factors that make older people more likely to suffer from malnutrition are social isolation and economic constraints. These and other factors put the elderly at risk of malnutrition. Additionally, ESKD which is an independent risk factor of nutritional impairment, in combination with older age, means that elderly people on hemodialysis should be given special emphasis in the context of diet and preventing the development of malnutrition. Some authors underline that good quality of life is very important in this group. The quality of food and physical fitness are inherent in the overall quality of life [21]. 

We obtained similar results as in the study by Celik et al., in which the elderly were characterized by a lower LTI value and a higher FTI [22]. Aging causes loss of muscle and bone mass and, on the other hand, an increase in fat mass. In addition, the distribution of adipose tissue changes in the elderly. An increase in intrahepatic and intra-abdominal fat has been observed in this group, which is conducive to metabolic disorders [23]. Sabatino et al. observed that low skeletal muscle area is associated with all-cause mortality in patients on hemodialysis [24].

In research by Barakat et al., the highest consumption of kilocalories was observed on dialysis days, while the lowest was observed on the day after dialysis [25]. Martins et al. observed that elderly patients on hemodialysis had lower dietary quality and higher consumption of processed foods than healthy elderly people. Interestingly, in this group of patients, the quality of the diet was lower on dialysis days than on days without dialysis [26]. The same team of scientists, in another study, showed that elderly people on dialysis days had lower intakes of energy, protein, lipids, potassium, and phosphorus than on days without hemodialysis [5]. A reduction in energy consumption on dialysis days was also observed in a multicenter study involving 1901 hemodialysis patients from 15 clinical centers across the United States [11]. Stark et al. showed differences between days in the amount of food intake. Patients on dialysis days consumed fewer grams of food than on non-dialysis days of the week and non-dialysis weekend days [27].

Differences between results may be due to various reasons. Eating habits differ between countries and other cultures. Even slight differences may appear with a local change, for example, between centers in the same area or even in the same clinic but under the care of other specialists. The diet and nutritional status also depend on economic conditions. As a rule, the economic conditions of the population improve over time. As time passes and more research results come in, scientific knowledge and recommendations evolve [28]. Conflicting results may be due to the notable time difference between studies. One of the goals of our study was to update and organize the knowledge about the nutrition of hemodialysis patients.

In 2021, Saglimbene et al. examined hemodialysis patients from 10 European countries. By the use of the frequency food questionnaire, the consumption of each product group and the nutrient content was calculated and compared with the recommendations in this group of patients. The data from the FFQ questionnaire reveals that vegetables, fruit, and cereals were the most often consumed. On the other hand, white and red meat, meat products, legumes, and nuts are the least frequent. The conclusion was that the diet was inconsistent with the recommendations [10]. In our study, the most often consumed products were vegetables, fruit, butter, and refined bread. The least frequent were venison, energy drinks, vodka, and avocado. Fruit and vegetable consumption levels are similar to Saglimbene’s studies [10].

Over time, the method of treating patients is developing along with new scientific reports. Not long ago, in 2007, the recommendation of the European Best Practice Guideline (EBPG) group stated that eating during or immediately before hemodialysis should be prohibited. The authors report that eating during a dialysis session may contribute to splanchnic vasodilation and thus lead to intra-dialytic hypotension [29]. In the statement from the ISRNM in 2018, it was mentioned that nutritional support in the form of a meal during dialysis should be standard, common practice in treatment centers [3]. 

On the one hand, intradialytic meals increase intradialytic blood pressure variability and lower the clearance of urea during dialysis [30]. Conversely, meals during dialysis sessions are documented to protect against PEW, reduce inflammation, and improve health-related quality of life [31].

Despite the growing number of scientific publications emphasizing the fact that the benefits of nutrition during dialysis are more substantial than the risk, especially in malnourished patients, there is still no clear answer as to whether intradialytic meals should be administered to patients. 

There are centers where meals during dialysis are not introduced and are even discouraged. From a survey conducted at the ISRNM Conference, in which nephrology specialists present at the conference were asked about their centers’ practices regarding meals during dialysis. Representatives of 73 clinics participated in the survey, 85% of clinics allowed patients to eat during dialysis, 65% actively encouraged them to do so, and 73% provided meals during treatment. Clinics that forbid eating during dialysis were in the minority (the authors did not provide exact percentages). This data is from 2014; any more recent data which describe the nutritional strategies of clinical centers during dialysis sessions have not been found [32]. 

Some believe that due to the risk of adverse effects caused by eating during dialysis, dialysis meals should not be widely encouraged, but the emphasis should be placed on adequate nutrition during the intervals between dialysis sessions [33]. However, the recommendation to increase energy supply is not easy to put into practice in hemodialysis patients. The nutrition recommended for patients with end-stage renal failure treated with dialysis is one of the most difficult to attend. The diet of these patients is restrictive, sometimes monotonous, and unpalatable to patients. Scrupulous adherence to dietary recommendations reduces the quality of life of patients. Additionally, dietary recommendations are complicated and may lead the patient into dissonance [10]. 

**Figure 2:**
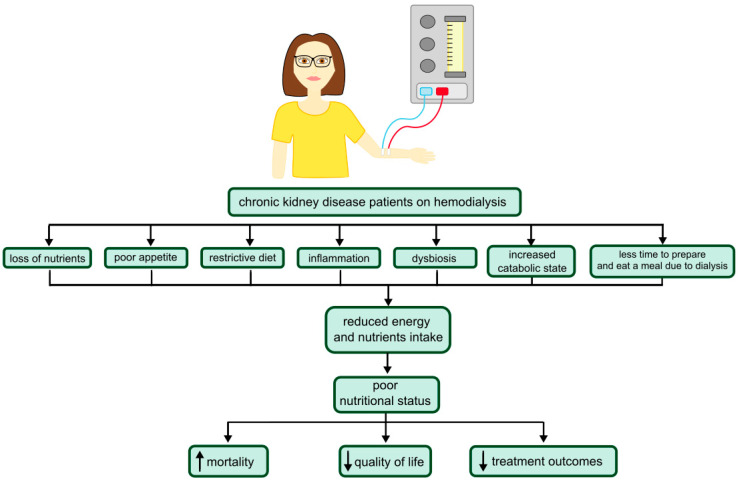
Factors leading to a reduction in energy and nutrient intake and its consequences in hemodialysis patients.

Patients undergoing renal replacement therapy poorly adhere to dietary recommendations, and their consumption of individual nutrients is not within the norms appropriate to their disease [34]. Lack of appetite, common in patients with CKD, is one of the manifestations of uremia. However, an increased dose of dialysis does not improve appetite. It can be explained by the fact that after the dialysis session, a post-dialysis fatigue effect occurs, which reduces the appetite [35]. Additionally, during dialysis sessions, patients have increased resting energy expenditure compared to the no-treatment period. [36]. Consequences of CKD, such as the accumulation of uremic toxins, dietary restrictions on fiber-rich foods, or the use of certain medications, may be involved in the development of CKD-related dysbiosis. By itself, an imbalance in the gut microbiota can cause disease progression and an increased risk of cardiovascular disease. Dysbiosis due to an insufficient amount of mucin as a lubricant causes less sliding of the fecal contents against the intestinal walls, which increases the transit time. In addition, altered bacterial fermentation plays a role in intestinal dysmotility. These phenomena lead to constipation, which is a common problem in hemodialyzed patients and significantly reduces their quality of life. Gastrointestinal symptoms, including constipation, can aggravate the reluctance to eat [37]. Another explanation for hemodialysis patients’ poor adherence to recommended nutrition and poor nutritional status is dysgeusia–- abnormal taste [38]. Factors leading to a reduction in energy and nutrient intake and their consequences are presented in Figure 2.

Even in the case where patients know the nutritional recommendations perfectly and have knowledge of which products contain particular ingredients and understand the nature of the disease and are able to adapt their diet to the current biochemical results, it may be difficult for patients to follow these recommendations due to the reasons mentioned above. Due to the inconclusive research results and the lack of consistent recommendations in the context of eating meals during dialysis, it is crucial to approach patients individually and consider the benefits and risks of an interdialytic meal in a specific case.

One of the most common causes of death in the population of hemodialysis patients is cardiovascular disease. This group of patients has a higher risk of these conditions due to inflammation, oxidative stress, accumulation of uremic toxins, and others. Additionally, the diet of patients with ESKD is a factor that may increase cardiovascular risk [39]. Dietary restrictions regarding the restriction of potassium and phosphate intake make the diet of hemodialysis patients far from the principles of nutrition in the prevention of cardiovascular diseases. Limiting the consumption of vegetables, fruits, legumes, and nuts is associated with a lower anti-inflammatory index [40,41].

Nuts, through the health-promoting ingredients they contain, including unsaturated fatty acids, fiber, vitamins, minerals, phenols, and phytosterols, reduce oxidative stress and inflammation. This improves the condition of blood vessels, insulin resistance, and lipid disorders, which in turn leads to beneficial effects on many diseases, including cardiovascular diseases. On the other hand, patients who consumed nuts more than once a day consumed more calories, protein, mono- and polyunsaturated fatty acids as well as phosphorus and potassium compared to those who eat nuts less frequently [42].

Currently, it seems that a reasonable amount of nuts—a handful a day (40 g) in hemodialysis patients—is safe and benefits the health of the patient [43]. Our results show that patients hardly ever eat nuts and seeds, which may increase cardiovascular risk.

The results of our study indicate that patients, especially the elderly, on dialysis days consume fewer calories and less protein. In the case of our patients, the reason for the reduction in caloric and protein intake on dialysis days should be determined. Is it a reduction in the willingness to eat due to the anorectic effect of dialysis, a lack of access to a meal if it is not arranged beforehand, or other reasons? The patients in our study are free to eat. Meals during dialysis are not provided, nor are they discouraged. The duration of dialysis is a great opportunity to consult a dietitian, establish a nutritional strategy and determine whether a meal introduced during dialysis would be beneficial for the patient, considering the nutritional status and diet of the patient. Constant specialist care by a dietitian is necessary for this group of patients. Firstly, to refresh patients’ knowledge about dietary recommendations and possible adjustments to the recommendations according to the patient’s condition. Secondly, to check the adherence to dietary recommendations and identify any difficulties. Another essential point is to make the patient aware of the importance of adhering to dietary recommendations and maintaining the patient’s motivation. 

Establishing habits is essential for proper functioning. Habitual behavior occurs without the use of cognitive processes, which allows you to perform various activities simultaneously and function properly in an environment full of various stimuli. On the other hand, if specific habitual behaviors are harmful, they can lead to health disorders. It is difficult to deactivate once developed habits. It is possible but requires cognitive effort [44]. It is well known that cognitive functions deteriorate with age [45]. As a result, older people may find it harder to change than younger people. For hemodialysis patients, it is necessary to change not only eating habits but also habits related to physical activity, which is necessary for the growth of muscle mass. Low LTI is associated with poorer long-term outcomes [46]. Changing habits, including eating habits and those related to physical activity, is a long and difficult process. Regular visits to dietitians and physiotherapists would help patients achieve their goals, which would positively affect their quality of life and improve their prognosis.

Our study has limitations. A 3-day food diary of a dialysis day, a non-dialysis day, and a weekend day is likely to show the patients’ usual diet. Despite the additional 24 h diet recall, asking questions about the consumption, and preparation of meals, we are not sure whether the diaries have been honestly completed. On the one hand, the fact that all three centers participating in the study are located close to each other, i.e., in Poland in the Pomeranian Voivodeship, may be a limitation of our study because it does not apply to the general population of hemodialysis patients in the world. On the other hand, as mentioned earlier, eating habits are shaped by the local environment; hence, studying a small geographical area will be associated with more detailed information about a specific group of patients, which will allow for the creation of a nutritional support strategy tailored to them. 

## 5. Conclusions

In HD patients, PEW develops frequently, which adversely affects survival. Sarcopenia and the risk of weakness syndrome may predominate in the elderly group. Patients’ diets do not meet their energy and protein requirements. Food consumption is lower on dialysis days than on other days. Nutritional education and the determination of whether meals are allowed during hemodialysis are essential to improving a patient’s nutrition and preventing geriatric syndrome.

## Figures and Tables

**Table 1:** Characteristics of the study group.

Parameters	All HD Patients *n* = 179	Age < 65 *n* = 74	Age ≥ 65 *n* = 105	*p*
Female/Man	78/101	33/41	45/60	0.82
Age (years)	64.6 ± 15.9 (68)	49.2 ± 11.5 (50.5)	75.5 ± 7.1 (74)	<0.01
Dialysis vintage (months)	45.0 ± 43.5 (31)	44.0 ± 43.5 (33)	45.9 ± 44.1 (30.5)	0.85
Kt/V	1.6 ± 0.35	1.61 ± 0.36	1.59 ± 0.34	0.93
Ultrafiltration (mL)	2146 ± 1080.5	2159 ± 1256.5	2135.7 ± 929.9	0.69
nPCR (g/kg)	0.94 ± 0.22	0.94 ± 0.2	0.94 ± 0.23	0.72

Data is presented as mean ± SD and (median) if the distribution is not normal. nPCR—normalized protein catabolic rate.

**Table 2:** Results of nutritional assessment.

Parameters	All HD Patients *n* = 179	Age < 65 *n* = 74	Age ≥ 65 *n* = 105	*p*
Body mass (kg)	74.5 ± 18.0 (72.5)	74.1 ± 20.3 (70.7)	74.9 ± 16.3 (72.8)	0.51
BMI (kg/m^2^)	25.4 ± 5.1 (25.1)	24.7 ± 5.4 (23.7)	26.0 ± 4.9 (25.8)	0.1
7-point SGA (points)	5.6 ± 1.0 (6)	5.5 ± 1.1 (6)	5.6 ± 0.9 (6)	0.47
LTI (kg/m^2^)	11.8 ± 3.3 (11.1)	12.3 ± 2.8 (12.5)	11.5 ± 3.7 (10.8)	0.01
FTI (kg/m^2^)	13.8 ± 5.9 (13.2)	12.5 ± 5.7 (11.7)	14.7 ± 6.0 (14.3)	<0.01
TBW (L)	33.8 ± 7.8 (32.2)	33.1 ± 8.5 (31.5)	33.6 ± 7.2 (32.5)	0.45
ECW (L)	15.8 ± 3.7 (15.2)	15.4 ± 4.1 (14.8)	16.1 ± 3.4 (15.5)	0.08
ICW (L)	17.4 ± 4.6 (16.6)	17.9 ± 4.5 (17.4)	17.0 ± 4.6 (16.0)	0.12
BCM (kg)	17.8 ± 7.3 (16.0)	19 ± 6.6 (19.4)	16.9 ± 7.6 (15.1)	0.02

Data is presented as mean ± SD and (median) if the distribution is not normal. BMI—body mass index, SGA—subjective global assessment, LTI—lean tissue index, FTI—fat tissue index, TBW—total body water, ECW—extracellular water, ICW—intracellular water, BCM—body cell mass.

**Table 3:** Comparison of dietary energy intake on a weekday with dialysis, a weekday without dialysis, and a weekend day without dialysis.

Dietary Energy Intake kcal/Day (kcal/kg/Day)							
Variable	*n*		Average Content over 3 Days	Weekday with Dialysis	Weekday without Dialysis	Weekend Day without Dialysis	*p*-Value *
All HD patients	118	mean ± SD	1483.5 ± 485 (23.7 ± 8.6)	1345.5 ± 539.4 (21.4 ± 9.1)	1523.5 ± 537.8 (24.2 ± 9.1)	1581.6 ± 485 (25.3 ± 9.8)	<0.01
		median	1434.1 (22.4)	1290.1 (19.5)	1462.8 (23.1)	1490.7 (23.5)	
		range	649.5–3179.1 (10–58.4)	275.3–4014.3 (5.8–54.2)	484.8–3131.1 (7.8–67.3)	694.7–545.6 (9.8–57.1)	
Classification by gender							
Women	50	mean ± SD	1325.2 ± 371.9 (24.1 ± 8)	1172.2 ± 423.8 (21.4 ± 9.1)	1349.7 ± 453.2 (24.3 ± 8.5)	1453.8 ± 431.2 (26.5 ± 9.5)	<0.01
		median	1293.5 (22.9)	1083 (19.1)	1223 (23.3)	1416.3 (25.6)	
		range	649.5–2035.6 (10–51.5)	275.3–2024.9 (6.9–52.5)	484.8–453.2 (7.8–46.1)	694.7–2402.1 (10–51.5)	
Men	68	mean ± SD	1600 ± 526.4 (23.4 ± 9.1)	1476 ± 581.3 (20.1 ± 9.3)	1651.3 ± 561.9 (24.2 ± 9.9)	1675.7 ± 602.3 (24.5 ± 10.1)	<0.01
		median	1519.7 (21.6)	1422.4 (20.1)	1558.7 (22.9)	1593.4 (22.6)	
		range	802.3–3179.1 (10.6–58.4)	405.5–4014.3 (5.8–54.2)	646–3131.1 (9.8–67.3)	741.2–3317.7 (9.8–57.1)	
Classification by 7-point SGA							
Well-nourished (SGA > 5)	73	mean ± SD	1516.9 ± 503.4 (23.2 ± 7.7)	1386.1 ± 592.2 (21.2 ± 8.9)	1556.9 ± 524.5 (23.8 ± 8.1)	1607.7 ± 546.7 (24.7 ± 8.6)	<0.01
		median	1443.6 (22)	1367.9 (19.3)	1513.7 (22.8)	1579 (23.4)	
		range	802.3–3179.1 (10.6–48.2)	405.5–4014.3 (5.8–54.2)	744–2786.9 (9.8–46.1)	694.7–3132.3 (9.8–50.8)	
Malnourished (SGA ≤ 5)	45	mean ± SD	1429.5 ± 453.8 (24.4 ± 9.9)	1279.8 ± 439 (21.9 ± 9.7)	1469.2 ± 560.3 (24.9 ± 11)	1539.4 ± 547.4 (26.5 ± 11.6)	<0.01
		median	1360.6 (22.4)	1256.1 (19.7)	1417.6 (23.4)	1418.8 (23.6)	
		range	649.5–2874 (10–58.4)	275.3–2494.5 (6.9–53.6)	484.8–3131.1 (7.8–67.3)	845.8–3317.7 (12.6–57.1)	
Classification by age							
Age < 65	50	mean ± SD	1640.3 ± 527.3 (26 ± 9.8)	1517.4 ± 620.4 (24 ± 10.6)	1699.5 ± 545.1 (27 ± 10.3)	1718.3 ± 624.9 (27.4 ± 11.4)	<0.01
		median	1544.9 (24.5)	1474 (22.8)	1555.4 (26)	1501.6 (25)	
		range	769.3–3179.1 (12.5–59)	275.3–4014.3 (6.9–54.2)	884.7–3131.1 (14.4–68.1)	694.7–3132 (10.1–54.8)	
Age ≥ 65	68	mean ± SD	1364.4 ± 403.5 (21.3 ± 6.5)	1220.2 ± 427.2 (19.1 ± 6.9)	1391.9 ± 482.5 (21.7 ± 7.3)	1479.9 ± 451.4 (23.2 ± 7.5)	<0.01
		median	1330.4 (20.4)	1166.7 (17)	1344.1 (21)	1430.5 (22.3)	
		range	649.5–2874 (9.8–41.6)	405.5–2213.3 (5.6–36.6)	484.8–3118.2 (7.6–45.4)	741.2–3317.7 (9.8–44.4)	

*p*-value *—weekday with dialysis vs. weekday without dialysis vs. weekend day without dialysis.

**Table 4:** Comparison of dietary protein intake on a weekday with dialysis, a weekday without dialysis, and a weekend day without dialysis.

Dietary Protein Intake g/Day (g/kg/Day)							
Variable	*n*		Average Content over 3 Days	Weekday with Dialysis	Weekday without Dialysis	Weekend Day without Dialysis	*p*-Value *
All HD patients	118	mean ± SD	65.8 ± 22 (1.04 ± 0.33)	58 ± 23.3 (0.91 ± 0.34)	68 ± 26.2 (1.07 ± 0.4)	71.4 ± 27 (1.13 ± 0.43)	
		median	62.6 (0.99)	55.3 (0.89)	63.6 (1.05)	67.4 (1.08)	<0.01
		range	29.6–146 (0.49–1.91)	13.3–134.1 (0.24–1.72)	15.9–161.4 (0.31–2.08)	28.4–154.5 (0.41–2.97)	
Classification by gender							
Women	50	mean ± SD	57.3 ± 16 (1.03 ± 0.31)	48.8 ± 17.1 (0.88 ± 0.33)	59.9 ± 20.6 (1.08 ± 0.39)	63.3 ± 21.1 (1.14 ± 0.39)	<0.01
		median	54.1 (0.98)	45.7 (0.87)	59.3 (1.05)	59.9 (1.19)	
		range	29.6–94.3 (0.49–1.68)	13.3–88.1 (0.33–1.59)	15.9–110.5 (0.31–2.08)	28.4–111.7 (0.49–2.23)	
Men	68	mean ± SD	72 ± 23.8 (1.04 ± 0.34)	64.8 ± 25 (0.93 ± 0.34)	74 ± 28.4 (1.07 ± 0.41)	77.3 ± 29.3 (1.12 ± 0.47)	<0.01
		median	69.8 (0.99)	60.2 (0.89)	68.1 (1.01)	70.6 (1.04)	
		range	38.1–146 (0.51–1.91)	16.6–134.1 (0.24–1.72)	37.8–161.4 (0.48–2.08)	31–154.5 (0.41–2.97)	
Classification by 7-point SGA							
Well-nourished (SGA > 5)	73	mean ± SD	69.1 ± 23.2 (1.05 ± 0.32)	61.2 ± 25.3 (0.93 ± 0.35)	71.4 ± 27.7 (1.08 ± 0.39)	74.7 ± 26.3 (1.14 ± 0.38)	<0.01
		median	66.5 (0.99)	55.6 (0.88)	65.5 (1.06)	69.7 (1.14)	
		range	31.1–146 (0.51–1.8)	16.6–134.1 (0.24–1.72)	23.8–161.4 (0.4–2.08)	28.4–149.1 (0.42–2.03)	
Malnourished (SGA ≤ 5)	45	mean ± SD	60.4 ± 19.1 (1.02 ± 0.35)	52.8 ± 18.8 (0.88 ± 0.31)	62.5 ±22.8 (1.05 ± 0.41)	66 ± 27.4 (1.12 ± 0.51)	0.02
		median	55.9 (0.95)	52.4 (0.9)	61.1 (1.02)	59 (1.03)	
		range	29.6–122 (0.49–1.91)	13.3–103.8 (0.28–1.45)	15.9–126.1 (0.31–2.01)	30.8–154.5 (0.41–2.97)	
Classification by age							
Age < 65	50	mean ± SD	68.4 ± 25.6 (1.07 ± 0.37)	61.2 ± 26.8 (0.95 ± 0.38)	71.1 ± 28.7 (1.11 ± 0.42)	73.8 ± 30.3 (1.16 ± 0.48)	<0.01
		median	64.3 (1.08)	54.5 (0.93)	64.6 (1.09)	67.2 (1.08)	
		range	29.7–146 (0.5–1.83)	13.3–134.1 (0.28–1.72)	23.6–161.4 (0.31–2.03)	31.5–154.5 (0.41–2.84)	
Age ≥ 65	68	mean ± SD	63.8 ± 18.7 (1.0 ± 0.29)	55.4 ± 19.8 (0.86 ± 0.29)	65.9 ± 23.8 (1.03 ± 0.37)	69.5 ± 24.03 (1.08 ± 0.37)	<0.01
		median	62.5 (0.96)	55.3 (0.87)	63.4 (1.02)	67.6 (1.07)	
		range	29.6–122 (0.48–1.63)	16.6–107.9 (0.23–1.5)	15.9–141 (0.3–2.07)	28.4–136 (0.41–2.06)	

*p*-value *—a weekday with dialysis vs. a weekday without dialysis vs. a weekend day without dialysis.

**Table 5:** The frequency of consumption of selected food products—(1) never or almost never, (2) once in a month or less, (3) several times a month, (4) several times a week, (5) daily, (6) several times a day.

Food Product	The Most Frequently Given Answer (Median)		The Percentage of Respondents Who Chose (5) or (6) Answers (i.e., the Percentage of People Consuming the Food Product at Least Once a Day)	
	Age < 65 *n* = 61	Age ≥ 65 *n* = 86	Age < 65 *n* = 61	Age ≥ 65 *n* = 86
Sugar	1 (3)	1 (2)	31.1	30.2
Honey	1 (2)	1 (2)	3.3	5.8
Chocolate, chocolate sweets	3 (3)	3 (3)	11.5	3.5
Non-chocolate sweets	3 (3)	1 and 4 (3)	9.8	15.1
Biscuits, cakes, muffins	3 (3)	3 (3)	8.2	14
Milk and natural milk drinks	3 (3)	4 (4)	29.5	25.6
Cheese	4 (4)	4 (3)	14.8	10.5
Eggs	3 (3)	4 (3)	0	5.8
Refined bread	5 (5)	5 (5)	54.1	52.3
Wholemeal bread or with grains	1 (3.5)	1 (3)	31.1	26.7
Oil	4 (4)	4 (4)	13.1	8.1
Butter	5 (5)	5 (5)	73.8	74.4
Fruit	4 and 5 (4)	5 (4)	42.6	46.5
Vegetables	5 (5)	5 (5)	55.7	54.7
Nuts	1 (2)	1 (1)	0	2.3
Seeds	1 (1)	1 (1)	0	1.2
Processed red meat (sausages)	3 (3)	4 (4)	14.8	19.8
High-quality cured meats (ham)	4 (4)	4 (4)	16.4	20.9
Red meat	3 and 4 (3)	3 (3)	4.9	0
White meat	4 (4)	4 (4)	4.9	1.2
Lean fish	3 (2)	3 (3)	1.6	0
Fat fish	2 (2)	3 (2)	0	0
Fruit juices, nectars	1 (3)	1 (1)	4.9	4.7
Sweetened beverages	2 (2)	1 (1)	8.2	0
Beer	1 (1)	1 (1)	0	1.2

The average calorific value of the diet was correlated with the frequency of consumption of honey (*p* < 0.05; R = 0.25), chocolate products (*p* < 0.05; R = 0.2), and nuts (*p* < 0.05; R = 0.22). The mean protein intake was correlated with the frequency of consumption of natural cottage cheese (*p* < 0.05; R = 0.19).

**Table 6:** Laboratory parameters in study groups.

Parameters	All HD Patients *n* = 179	Age < 65 *n* = 74	Age ≥ 65 *n* = 105	*p*	References Values
Hemoglobin (g/dL)	10.6 ± 1.3	11.0 ± 1.3	10.4 ± 1.1	<0.01	9.5–12.5
BUN before HD (mg/dL)	50.7 ± 14.0	51.4 ± 13.8	50.0 ± 14.3	0.57	8.4–25.7
BUN after HD (mg/dL)	13.6 ± 5.7 (13)	13.7 ± 6.3 (13.0)	13.4 ± 5.3 (13.0)	0.85	8.4–25.7
Transferrin (g/L)	1.78 ± 0.64 (1.7)	1.73 ± 0.31 (1.71)	1.82 ± 0.82 (1.67)	0.97	1.8–3.91
Total cholesterol (mg/dL)	163.6 ± 46.3 (159)	169.7 ± 45.4 (165.5)	158.5 ± 46.8 (158)	0.19	<190
Albumin (g/L)	36.1 ± 6.4 (35.5)	36.6 ± 5.2 (37)	35.7 ± 7.2 (35)	0.19	38–48
CRP (mg/dL)	12.5 ± 24.7 (4.95)	13.2 ± 27.4 (6.1)	14.2 ± 25.6	0.63	<5
PTH (pg/mL)	614.6 ± 742.6 (375)	873.5 ± 973 (497.5)	397 ± 351 (305)	<0.01	11–67

Data is presented as mean ± SD and (median) if the distribution is not normal. BUN—blood urea nitrogen, HD—hemodialysis, CRP—C-reactive protein, PTH—parathyroid hormone.

**Table 7:** Relationship between nutritional status, body composition, and biochemical parameters.

	Body Weight (kg)	BMI (kg/m^2^)	7-Points SGA	LTI (kg/m^2^)	FTI (kg/m^2^)
Albumin (g/L)	0.24 *p* = 0.026	0.17 *p* = 0.11	0.25 *p* = 0.025	0.14 *p* = 0.2	0.11 *p* = 0.23
BUN before HD (mg/dL)	0.21 *p* = 0.04	0.17 *p* = 0.12	0.09 *p* = 0.4	0.19 *p* = 0.08	0.06 *p* = 0.53
BUN after HD (mg/dL)	0.53 *p* < 0.01	0.49 *p* < 0.01	0.19 *p* = 0.08	0.32 *p* = 0.003	0.13 *p* = 0.19
Total cholesterol (mg/dL)	0.11 *p* = 0.17	0.19 *p* = 0.04	0.14 *p* = 0.15	−0.18 *p* = 0.059	0.29 *p* = 0.002
CRP (mg/dL)	−0.04 *p* = 0.64	−0.13 *p* = 0.18	−0.3 *p* = 0.002	−0.03 *p* = 0.74	−0.15 *p* = 0.13

BUN—blood urea nitrogen, HD—hemodialysis, CRP—C-reactive protein, BMI—body mass index, SGA—subjective global assessment, LTI—lean tissue index, FTI—fat tissue index.

## Data Availability

Not applicable.
